# 

**Published:** 1961-07

**Authors:** 


### Contents

SOME LESSER-KNOWN ACTIVITIES OF THE GENERAL MEDICAL COUNCIL . . . . . . *Professor R. J. Brocklehurst* 75

REFLECTIONS ON THE INDICATIONS FOR PROSTATECTOMY *J. P. Mitchell* 85

A CASE OF UNILATERAL INVOLUNTARY MOVEMENTS TREATED WITH PROCAINE AMIDE . . . . . *W. Cullen and A. Leitch* 92

A COAGULATION DEFECT IN AMYLOIDOSIS *I. D. Fraser and M. R. Wills* 97

NOTES AND NEWS . . . . . . . . 101

APPOINTMENTS . . . . . . . . . 101

